# Evolution of viral genomes and their clouds of sequence

**DOI:** 10.1073/pnas.2525457122

**Published:** 2025-12-22

**Authors:** Karla Kirkegaard

**Affiliations:** ^a^Violetta L. Horton Professor, Departments of Genetics and of Microbiology and Immunology, Stanford University School of Medicine, Stanford, CA 94305

When a novel virus such as Sudden Acute Respiratory Syndrome Coronavirus 2 (SARS-CoV-2) infects a new host species, it faces an inhospitable environment. However, a few mutations can be all the virus needs to get a bit of replication accomplished. As we know from the SARS-CoV-2 pandemic, the results of adaptation in even a few hosts can be catastrophic for the entire new host population. The remarkable paper by Martínez-González et al. (1) has documented both the rate of viral population change and the sizes of the quasispecies clouds that accompanied the waves of SARS-CoV-2 infection as they reached one metropolitan area, Madrid, during the first years of the pandemic (1).

## What Is a Quasispecies Cloud?

Following its introduction into the human population in 2019, the approximately 30,000-nucleotide SARS-CoV-2 genome has evolved at a relatively constant rate of 10^−3^ to 10^−4^ substitutions per nucleotide each year (1; figure 2). These mutations remained in the population via genetic drift or via selective pressure from the millions of variants produced. Within each infected cell, the viral RNA replication machinery makes errors at a frequency such that each templating event gives rise to approximately one mutation per genome. Mutations accumulate because multiple positive-to-negative and negative-to-positive templating events are needed to create the thousands of viral RNAs in each infected cell. The iterative and cumulative nature of mutation acquisition results in the structure of the “quasispecies cloud” in each infected cell and ultimately in each infected person.

The size of this cloud in an infected individual will depend on the viral RNA load, the intrinsic mutation frequency of the viral polymerase complex, and the number of templating steps required to create infectious virions in the infected cell types. Unless these parameters change, the polymerase mutation frequency and quasispecies cloud size should remain proportionate.

## The Quasispecies Clouds Have Decreased in Size during the SARS-CoV-2 Pandemic

The experiments described by Martínez-González et al. have documented both the rate of consensus-sequence change and the size of the quasispecies clouds that accompanied seven waves of SARS-CoV-2 infection in Madrid as the internationally spreading virus arrived. Ten samples from each discrete wave were analyzed by deep sequencing. The authors were careful to select samples with no clear differences in disease severity or viral load. Interestingly, the consensus sequence of each sample in any given wave was unique. The cloud size for each sample was determined by evaluating the amount of sequence space explored surrounding the consensus sequence of each sample, corrected by sample size (2).

Significant reduction in the in the sizes of the quasi-species clouds was observed after the first wave of infection (1; figure 3). This decrease in mutant spectrum complexity was observed in both the rapidly evolving Spike-coding sequence and the RNA-dependent RNA polymerase (nsp12) coding sequences, whose consensus sequence evolved more slowly. This argues that a genome-wide decrease in mutational complexity occurred as the virus evolved in the human population.

How did this happen? The first, most likely possibility for the shrinkage in mutational spectra as the pandemic progressed was that the virus evolved to reduce the misincorporation frequency of the viral RNA replication machinery. The authors excluded this possibility by passaging virus samples from Wave 1 and Wave 7 in cultured human cells. Under these circumstances, quasispecies clouds of comparable magnitude were observed, arguing that the intrinsic mutation frequency of polymerization had not altered between Wave 1 and Wave 7.

The authors thus favor a hypothesis that the virus changed its relationship with its host as it evolved. SARS-CoV-2 was introduced into the human population by a small number of transmission events from nonhuman hosts. The low frequency of these transmission events illustrates our good luck that species jumping can be difficult for a virus. To survive in the new host, the viral genome must have evolved to use the scores of human host proteins required for cell entry, uncoating, translation, RNA replication, packaging and subsequent spread. But why and by what mechanism did the virus decrease the amount of sequence space available to it during its adaptation to growth in humans? Perhaps the question is not: “Why is the complexity of the mutational cloud reduced in Waves 2 to 7?” but “Why was the mutational spectrum so complex in Wave 1?”

## “Mutant Spectrum Complexity May Be a Variable Trait in the Course of a Virus Pandemic”

This statement by the authors might have seemed anti-Darwinian in the past. However, a potential precedent can be found in stress-induced mutagenesis in bacteria. As shown by the laboratory of Susan Rosenberg and others, stress such as the addition of antibiotics not only results in selection of drug-resistant bacteria that appear at the expected mutation frequency but also as a consequence of induced mutation-prone repair and recombination pathways that lead to unexpectedly high survival (3–5).

The remarkable paper by Martínez-González et al. (1) has documented both the rate of viral population change and the sizes of the quasispecies clouds that accompanied the waves of SARS-CoV-2 infection as they reached one metropolitan area, Madrid, during the first years of the pandemic (1).

In the case of a positive-strand RNA virus such as SARS-CoV-2, one example of how adaptation to a new host environment could alter the mutational spectrum complexity is by changing the number of templating events required to generate new viral genomes. Positive- and negative-strand synthesis often require different host proteins, and alterations in templating patterns are predicted to alter the shape of the mutational spectrum (6). For example, a “stamping machine,” in which 10 negative strands template 100 positive-strand progeny each to give 1,000 viruses, will lead to the accumulation of many fewer mutations than a “geometric growth” mechanism, with many iterative rounds of positive- and negative-strand synthesis. Many other scenarios can be envisaged.

The surprising finding that viral quasispecies complexity can alter during and after adaptation to a new host alters our understanding of viral emergence. Whether the larger magnitude of the quasispecies cloud was due to stress responses that altered protein synthesis, to changes in iterative viral RNA templating or as-yet-unimagined mechanisms, understanding this new twist in viral evolution may inform antiviral design and pandemic management. It will certainly inspire increased precision in our discussions of the roles of polymerase misincorporation frequencies, quasispecies complexity and virus-host relationships as they, too, continue to evolve.
